# Yolk vitamin E prevents oxidative damage in gull hatchlings

**DOI:** 10.1098/rsos.170098

**Published:** 2017-05-31

**Authors:** Marco Parolini, Lela Khoriauli, Cristina Daniela Possenti, Graziano Colombo, Manuela Caprioli, Marco Santagostino, Solomon G. Nergadze, Aldo Milzani, Elena Giulotto, Nicola Saino

**Affiliations:** 1 Department of Environmental Science and Policy, via Celoria 26, I-20133 Milan, Italy; 2 Department of Biosciences, University of Milan, via Celoria 26, I-20133 Milan, Italy; 3 Department of Biology and Biotechnology, University of Pavia, via Ferrata 1, I-27100 Pavia, Italy

**Keywords:** maternal effects, vitamin E, oxidative stress, telomere, yellow-legged gull

## Abstract

Oxidative stress experienced during early development can negatively affect diverse life-history traits, and organisms have evolved complex defence systems against its detrimental effects. Bird eggs contain maternally derived exogenous antioxidants that play a major role in embryo protection from oxidative damage, including the negative effects on telomere dynamics. In this study on the yellow-legged gull (*Larus michahellis*), we manipulated the concentration of vitamin E (VE) in the egg yolk and analysed the consequences on oxidative status markers and telomere length in the hatchlings. This study provides the first experimental evidence that, contrary to the expectation, a physiological increase in yolk VE concentration boosted total antioxidant capacity and reduced the concentration of pro-oxidant molecules in the plasma, but did not reduce telomere attrition or ameliorate oxidative damage to proteins and lipids in the early postnatal period.

## 1. Introduction

Early-life development is characterized by rapid growth requiring high metabolic activity and oxygen consumption, which imposes notable reactive oxygen species (ROS) production [1], even if there is still ambiguity regarding the relationship between oxygen consumption and ROS production [2]. To efficiently counteract the detrimental effects of oxidizing molecules, organisms have evolved a complex antioxidant machinery, which relies on enzymatic and non-enzymatic defence. In oviparous species, embryo protection against ROS largely depends on egg exogenous non-enzymatic antioxidants of maternal origin [3]. However, a small amount of ROS may escape from the protective shield of antioxidants, causing an oxidative stress situation to organisms, which can lead to oxidative damage to cellular macromolecules, including lipids, protein and DNA [1]. Oxidative stress-related adverse effects can occur throughout an individual's life and influence diverse life-history traits, representing a constraint in many biological processes [4]. Only recently, oxidative stress has been shown to interfere with telomere dynamics [5,6]. In vertebrates, telomeres are conserved non-coding sequences of the repeated TTAGGG motif that cap the ends of chromosomes and protect genomic integrity [7]. Telomeres shorten with age, and short telomeres at birth or rapid telomere loss are associated with reduced performance at several fitness traits and survival [5,6]. Oxidative stress has been suggested to provide a potential mechanism for telomere attrition in early life, hastening cell senescence and leading to negative consequences on survival and fitness-related traits of the offspring [5,6]. As antioxidants can decelerate telomere shortening [8], maternal allocation of exogenous antioxidants to the egg yolk may contribute to the maintenance of telomere length (TL) during early development.

Maternal egg antioxidants can modulate offspring performance and phenotype according to complex ‘maternal effects’ pathways. Low levels of maternal yolk antioxidants impair embryo development, suggesting their pivotal role in the early defence against ROS [3]. Vitamin E (VE) is one of the most important maternally transferred yolk antioxidants and plays a fundamental role in ROS scavenging [3]. Experimental dietary administration of VE has been shown to have beneficial effects on diverse offspring traits and in the prevention of deleterious effects caused by ROS in chicks of captive and wild species [1]. Differently, the beneficial effect of VE supplementation on telomere dynamics in birds, mainly during the early-life period, is still largely unexplored, albeit expected. As oxidative stress accelerates telomere shortening [8], VE supplementation may prevent telomere shortening because of its antioxidant capacity [9]. Studies of humans and other vertebrates, but not in birds, have demonstrated the beneficial effects and the underlying mechanism of action of VE supplementation on telomeres. Shortening of telomeres was slowed down in human cells supplemented with physiological doses of VE, which reduced ROS production and limited oxidative damage to telomeric DNA [10], although Guan and coauthors [11] showed that VE supplementation did not positively affect TL in peripheral blood mononuclear cells from Alzheimer's disease patients. Larger dietary intake of VE has been found to be associated with longer telomeres in humans [12], and *in vitro* experiments on skin fibroblasts have demonstrated that VE restores telomerase activity and protects against telomere erosion [13], suggesting that the protective role of VE against ROS-induced DNA damage is mediated by the up-regulation of c-fos expression and AP-1-binding activity [13].

In this study of the yellow-legged gull (*Larus michahellis*), we assessed the effect of a physiological increase in yolk VE concentration on oxidative status markers (i.e. total antioxidant capacity, amount of pro-oxidant molecules, lipid peroxidation and protein carbonylation) and TL of the newly hatched chicks. We expect that VE supplementation positively affects oxidative status, reduces oxidative damage and results in longer TL in VE-treated chicks as compared to controls. As VE concentration declines with laying order [14] and limits the postnatal growth of hatchlings from the last-laid (typically third) eggs [15], we also expect a differentially larger positive effect of VE on chicks from third-laid eggs. Because no difference in the concentration of yolk VE in the yellow-legged gull according to the sex of the embryo occurs [14] but embryos of either sex may show different susceptibility to yolk antioxidants, we also tested if the effect of VE injection depended on the sex of the chicks.

## 2. Material and methods

The experiment was performed during March–May 2014 in a large breeding colony in the Comacchio lagoon (NE Italy, 44°20′ N–12°11′ E). Full details of the experiment are reported in [15] and in the electronic supplementary material. We aimed at increasing the yolk VE concentration (α- and γ-tocopherol mixture) by 1 standard deviation of that measured in eggs of gulls from the same colony [14] through a previously validated injection method. We adopted a within-clutch design whereby the VE dose due to be injected was tuned according to egg size at laying and position in the laying sequence. After VE injection, the nests were visited every day. At hatching a blood sample was collected for molecular sexing, oxidative status markers and TL analyses. Total antioxidant capacity (TAC) and the amount of pro-oxidant molecules (i.e. TOS) were measured according to colorimetric methods [16]. Protein carbonylation was assessed by western immunoblotting [16], while lipid peroxidation through the thiobarbituric acid reactive substances (TBARS) method [17]. It should be noted that the TBARS method may not measure oxidative damage to lipids accurately because TBA reacts with other compounds, apart from the main lipid peroxidation by-product malondialdehyde (MDA). Thus, TBARS results should be interpreted with caution because they may overestimate lipid peroxidation (LPO). TL was measured using the monochrome multiplex quantitative PCR method (MMQPCR) [18] and expressed as the ratio between the amount of telomeric repeats in the sample (T) and that of a single copy gene (S), relative to a reference sample (relative telomere length, RTL). All methods are fully described in the electronic supplementary material. We also verified the effectiveness of VE injection by measuring VE concentration in the yolk of some VE-injected eggs, which was always higher than that of sham-injected eggs (see electronic supplementary material for details). The effect of VE on investigated endpoints was analysed in linear mixed models (LMMs), including clutch identity as a random intercept effect. Egg treatment, embryo sex and egg-laying order were included as fixed-effect factors along with their two-way interactions. Egg mass at laying was included as a covariate in all models. All non-significant (*p* > 0.05) interaction terms were removed from the models in a single step. The effect of clutch identity was tested by the likelihood ratio test. Five chicks could not be sexed and were therefore excluded from all the analyses. Oxidative damage and TL analyses could not be assessed in some (2–9) hatchlings. In all the analyses, we always used the largest sample available. Statistical analyses were performed by using SAS 9.3 PROC MIXED. Statistics are presented as estimated marginal means (EMMs) ± standard error (SE).

## 3. Results

**Figure 1.**
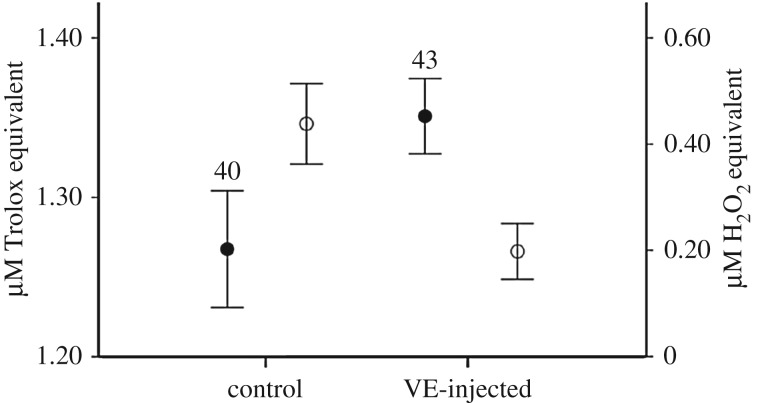
Estimated marginal mean (±95% confidence intervals) of TAC (µM Trolox equivalent; black circles) and TOS (µM H_2_O_2_ equivalent; white circles) measured in the plasma of hatchlings from control and VE-injected eggs. Sample sizes are reported.

An LMM showed that VE treatment caused a statistically significant increase in TAC in the hatchlings from VE-injected eggs compared to controls (EMM: controls: 1.27 (0.02); VE-treated: 1.36 (0.02); figure 1). Sex and laying order did not significantly predict TAC of the hatchlings (table 1). TOS levels were significantly lower in the plasma of chicks hatched from VE-injected eggs with respect to controls (control eggs: 0.429 (0.03); VE-injected eggs: 0.192 (0.03); figure 1). Both TAC and TOS significantly varied among broods (likelihood ratio test; $\chi_{1}^{2}>5.6,$ *p* < 0.017). No effect of VE treatment, sex, laying order and their interactions was found for protein carbonylation and lipid peroxidation in blood samples from hatchlings (table 1). Finally, VE supplementation did not significantly affect RTL, after controlling for the potentially confounding effects of sex and laying order (table 1). Separate LMMs of RTL where we included the markers of oxidative status as covariates did not reveal any significant effect (*F* < 2.04, *p* > 0.157 in all cases).

**Table 1.** Linear mixed models of total antioxidant capacity (TAC), amount of pro-oxidant molecules (TOS), lipid peroxidation (LPO), protein carbonylation (PCO) and relative telomere length (RTL) in the blood of yellow-legged gull hatchlings in relation to VE treatment, sex and laying order. Clutch identity was included in the model as a random intercept effect. The non-significant effects of the two-way interactions between fixed factors were excluded from the final model. C, control; VE, vitamin E-injected. Significant effects are reported in italics.

	TAC (C = 40; VE = 43)			TOS (C = 40; VE = 43)			PCO (C = 38; VE = 42)			LPO (C = 36 ; VE = 38)			RTL (C = 38; VE = 42)		
sample size	*F*	d.f.	*p*	*F*	d.f.	*p*	*F*	d.f.	*p*	*F*	d.f.	*p*	*F*	d.f.	*p*
final model															
treatment	25.06	1, 57	<*0*.*001*	36.14	1, 59	<*0*.*001*	2.24	1, 55	0.140	0.01	1, 50	0.927	0.13	1, 74	0.723
sex	0.39	1, 63	0.536	0.24	1, 65	0.629	0.31	1, 60	0.581	1.18	1, 46	0.282	1.88	1, 74	0.174
laying order	4.79	2, 59	*0*.*012*	1.69	2, 61	0.193	1.63	2, 59	0.205	0.43	2, 45	0.651	0.40	2, 74	0.670
excluded terms															
treatment × sex	0.46	1, 70	0.502	0.16	1, 71	0.692	0.05	1, 67	0.815	0.59	1, 46	0.445	0.02	1, 69	0.883
treatment × laying order	0.66	2, 67	0.522	1.14	2, 68	0.326	0.90	2, 64	0.413	0.13	2, 52	0.875	0.42	2, 69	0.656
sex × laying order	0.58	2, 66	0.565	1.28	2, 68	0.284	0.19	2, 62	0.831	0.17	2, 45	0.846	0.21	2, 69	0.810

## 4. Discussion

The experimental increase in yolk VE concentration within physiological limits ameliorated plasma TAC and TOS, but this was not mirrored in a reduction in oxidative damage to proteins and lipids. In addition, VE supplementation did not affect TL, contrary to the expectation, stemming from the hypothesis of a negative effect of pro-oxidants on TL.

VE supplementation significantly increased plasma TAC and reduced TOS, confirming its crucial antioxidant role (figure 1). Similar effects were found in the plasma of hen chicks supplemented via the diet with supra-physiological VE doses [19], but are not consistent with those found in great tit nestlings, where neither plasma TAC nor TOS differed between experimental groups after administration of VE-enriched food [20]. Although our previous studies showed that VE supplementation exerted positive effects on morphological traits of chicks hatched from third-laid VE-injected eggs [15], the significant effect on TAC and TOS was independent of egg laying order, suggesting that all chicks benefited from VE supplementation. However, we did not detect any effect of yolk VE increase on oxidative damage to proteins and lipids according to previous studies of wild birds [1,21]. Contrary to the expectation, VE treatment had no effect on TL in red blood cells, despite having positive effects on oxidative status. Oxidative stress has been often invoked as a determinant of telomere attrition, but no experimental study to date has capitalized on the advantages of the avian eggs as a cleidoic environment amenable to controlled manipulation of the level of antioxidants in the prenatal environment. While *in ovo* corticosterone injection caused ROS overproduction and telomere shortening in domestic chickens at 21 days [22], no experimental study of birds has tested for the effect of prenatal antioxidants on TL at the end of the embryonic stage, when telomere attrition is believed to have already progressed. These findings are the first experimental evidence that VE egg supplementation, mimicking physiological variation in maternal transfer to the egg, does not affect TL at hatching. Postnatal dietary supplementation of VE and vitamin C in the yellow-legged gull has also been shown to have no effect on TL of 7-day-old chicks [23]. These results combined suggest that availability of egg maternal and dietary VE has little influence on telomere dynamics in early life stages. However, such effects may become apparent at a later life stage, as shown for blue tit nestlings where the positive effect of a one-shot treatment with VE and methionine via subcutaneous injection on TL could be recorded 1 year after treatment [24].

Our study shows that a physiological increase in VE yolk concentration has positive effects in terms of plasma TAC and reduction in TOS but has no effect on oxidative damage or TL at hatching. This suggests that maternal allocation of VE to the egg is not limiting to protection from oxidative damage and any reduction of TL during prenatal life. However, we cannot exclude that the improvement of oxidative status of hatchlings due to the increase of VE concentration may result in positive effects in later life stages. Although TL at birth is considered an important predictor of fitness-related traits, telomere attrition can be more intense during postnatal growth. Thus, the availability of maternally transferred dietary antioxidants during early life may have long-term consequences by alleviating the costs of stressful conditions experienced during growth and preventing telomere attrition and the subsequent age-related risk factors for disease and increased risk of mortality.

## Supplementary Material

ESM_1

## Supplementary Material

ESM_2

## Data accessibility

The data supporting this article are in the electronic supplementary material.
