# Supplementary material for: Yolk vitamin E prevents oxidative damage in gull hatchlings
Source: R Soc Open Sci. 2017 May 31;4(5):170098. doi: 10.1098/rsos.170098 (PMC5451819; doi:10.1098/rsos.170098)
Supplement: ESM_1 [file rsos170098supp1.docx]

**Electronic Supplementary Material (ESM)**

**Yolk vitamin E prevents oxidative damage in gull hatchlings**

**Field procedures**

The present study was carried out on a large colony (> 400 breeding pairs) of yellow-legged gull (*Larus michahellis*) in the Comacchio lagoon (NE Italy) during March-May 2014. The colony was visited every second day to check for any new nests and newly laid eggs, which were marked to monitor the progress of laying and to identify laying sequence. When a new egg was found, it was temporarily removed from the nest for experimental manipulation while temporarily replacing it with a 'dummy' egg.

The experiment was performed as described in detail by Parolini et al. (2015). We aimed at increasing the concentration of vitamin E (VE; mixture of α- and γ-tocopherol; 93:7 ratio) by 1 standard deviation (SD) of that measured in the yolk of yellow-legged gull eggs from the same colony (Rubolini *et al.* 2011), by *in ovo* injection. After injection, the final concentration of VE was within the natural range of variation. Since the concentration of VE in the yolk varied according to egg size and position in the laying sequence, we tuned the dose due to be injected according to these factors. Therefore, based on Rubolini *et al.* (2011), we grouped first (a-), second (b-) or third (c-) laid eggs into three classes (tertiles) of size according to egg mass and we calculated the standard deviation of VE concentration in the yolk for each tertile within each position in the laying sequence (Table S1).

**Table S1**. Mean concentrations (± SD) of VE (µg/g) measured in yolk from yellow-legged gull eggs (data from Rubolini et al., 2011). First (a-), second (b-) and third (c-) laid eggs were grouped into three classes (tertiles) of size according to egg mass.

| **Mean VE concentration ± SD (µg/g)** | | | |
| --- | --- | --- | --- |
|  | **Tertile** | | |
| **Laying order** | 1st | 2nd | 3rd |
| a-egg | 115.1 ± 21.3 | 132.0 ± 22.1 | 124.0 ± 24.6 |
| b-egg | 102.3 ± 21.2 | 115.7 ± 22.5 | 102.2 ± 23.1 |
| c-egg | 73.5 ± 19.5 | 92.9 ± 21.6 | 88.9 ± 21.5 |

Then, we estimated yolk mass based on total egg mass for each class of position in laying sequence as follows: yolk mass = 0.227 (0.039 SE) egg mass + 1.815 (3.461 SE); R^2^ = 0.252; F_1,88_ = 34.38, P < 0.001). The amount of VE due to be injected was then calculated as the product of the relevant standard deviation value and yolk mass thus estimated. We adopted a within-clutch design, whereby both control and VE-injected groups were established within each clutch, to minimize the confounding effects of environmental and parental effects. The following treatment schemes were assigned sequentially to the clutches, according to the order in which the first egg was found (nest, a-, b-, c-egg): nest 1, VE injection (E), control injection (C), E; nest 2, C-E-C; nest 3, E-C-C; nest 4, C-E-E and so forth with the following nests. The amount of VE injected in the three classes of egg mass for the three positions in the laying sequence is reported in Table S2.

**Table S2**. Amount (µg) of VE (α : γ – tocopherol ratio *per* egg) injected into the yolk of yellow-legged gull eggs depending on egg mass at the time of deposition and laying order (first, second or third egg is a-, b-, or c-egg, respectively). The doses were designed to increase the post-manipulation vitamin E concentration of 1 standard deviation compared to that previously recorded in the same population for each class of egg mass and position in the laying sequence.

| *Laying order* | *Egg mass (g)* | *Vitamin E* (*µg*)  *(α : γ - tocopherol)* |
| --- | --- | --- |
| a-egg | 84-91 | 670 (623:47) |
|  | 92-95 | 748 (696:52) |
|  | 96-108 | 697 (648:49) |
| b-egg | 80-88 | 509 (473:36) |
|  | 89-92 | 699 (650:49) |
|  | 93-99 | 688 (640:48) |
| c-egg | 75-82 | 305 (283:22) |
|  | 82-87 | 616 (573:43) |
|  | 88-98 | 643 (596:45) |

VE solutions were prepared in sterile vials by dissolving α- and γ-tocopherol in corn oil to the final dilution required. Each vial contained the desired concentration of VE to be injected in egg yolk depending on egg mass and laying order. Treated eggs were injected with 30 µl of the appropriate concentration of VE, while control eggs were injected only with 30 µl of corn oil.

VE was injected in the yolk with the same procedure reported in Romano et al. (2008). Before being injected, the egg was weighed (to the nearest g) and placed with the longitudinal axis vertical. After disinfecting the eggshell, a hole was drilled using a sterile pin close to the acute pole. *In ovo* injection was performed by means of 1-mL sterile syringe mounting a 0.6 × 30 mm needle while the egg was held firmly with its longitudinal axis vertical. Immediately after extracting the needle from the egg, the hole was sealed with a drop of epoxidic glue and a small piece of eggshell superimposed to the hole.

We verified the effectiveness of VE injection by measuring VE concentration in yolk of some VE-injected eggs, which was always higher than that of sham-injected eggs (see details below).

After the *in ovo* VE injection, all the nests were visited every day and eggs were monitored until hatching. Because normally up to two days elapse between the time when the egg reaches the pipping stage and hatching, we assigned chicks to their original egg by injecting in the pipping egg a small drop of food dye (either blue or green; Bonisoli-Alquati et al., 2007). Upon the first daily visit to the nest when any individual chick was found to have hatched, the chick was weighed (to the nearest g) and its tarsus was measured (to the nearest 0.1 mm) (see Parolini et al. 2015). Finally, a blood sample (about 100 μl) was collected in capillary tubes after puncturing the ulnar vein. Blood samples were centrifuged at 11,500 rpm for 10 min to separate red blood cells from plasma, which were stored at – 20 °C until biochemical analyses. All the measurements were taken by the same person for consistency. Molecular sexing of embryos and chicks was performed according to Rubolini et al. (2006).

As reported by Parolini et al. (2015), mean clutch size was 2.86 (0.41 SD) eggs, with 39 (89%) of the clutches containing 3 eggs. Hatching success was very similar between the control, sham-injected (proportion of hatched eggs = 43/61 = 0.705) and the VE-injected (45/63 = 0.714; χ^2^_1_ = 0.01, P = 0.93) eggs. The sex ratio among the chicks that successfully hatched was also similar between experimental groups (proportion of males: sham-injected: 18/40 = 0.450; VE: 20/43 = 0.465; χ^2^_1_ = 0.01, P = 0.91). In a LMM on the eggs that successfully hatched, where clutch identity was included as a random effect, egg mass did not differ between the two experimental groups (F_1,49.5_ = 0.02, P = 0.894). In the same model, egg mass significantly declined with laying order (estimated marginal means (SE): first eggs: 91.4 (0.90); second eggs: 89.1 (0.90); third eggs: 83.8 (0.97)), with significant pairwise differences among all laying order groups (LSD test: P < 0.012 in all cases).

**Methods of Oxidative status markers**

Oxidative stress assays were performed in blood samples of chicks hatched from both control and VE-injected eggs. Total antioxidant capacity (TAC), amount of pro-oxidant molecules (TOS) and protein carbonyl content (PCO) were measure in plasma, while lipid peroxidation (LPO) was evaluated in red blood cells.

TAC was measured according to a colorimetric method developed by Erel (2004), with modifications. The color of 2,2’-azinobis-(3-ethylbenzothiazoline-6-sulfonic acid) radical cation (ABTS*+) bleaches depending on the concentration of antioxidants in the sample. The reaction is monitored spectrophotometrically and the final absorbance is inversely related to TAC of the sample. The assay was calibrated by drawing a standard curve with serial dilutions of Trolox and the results were expressed as µM Trolox equivalent. Mean TAC intra-assay coefficient of variation (CV) was 2.8 ± 0.5 % (n = 3 replicates), while the mean inter-assay CV was 6.9 ± 0.5 % (n = 3 assay plates).

TOS was measured according to a colorimetric method adapted from Erel (2005). The oxidants in the plasma oxidize the ferrous ion-*o*-dianisidine complex to the ferric ion, which reacting with xylenol orange gives a blue complex. Coloration was measured by a spectrophotometer at λ = 535 nm and is proportional to the oxidants in the plasma. The assay was calibrated by using a standard curve with serial dilution of hydrogen peroxide (H_2_O_2_). The results were expressed as µM H_2_O_2_ equivalents. The mean TOS intra-assay CV was 2.4 ± 1.2 % (n = 3 replicates) and the inter-assay CV was 4.1 ± 2.2 % (n = 3 assay plates).

Carbonylated proteins were derivatized with 2,4-dinitrophenylhydrazine (DNPH). Briefly, 400 μg proteins in 50 mM Tris-HCl pH 7.4 (final concentration 1 mg mL^-1^) were mixed with 80 μL of 10 mM DNPH in 2N HCl and incubated for 60 min in the dark with frequent vortexing. After derivatization, protein samples were mixed with 480 μL of 20% trichloroacetic acid (TCA) 20% and incubated for 10 min in ice. After centrifugation at 20,000 g for 15 min at 4 °C, protein pellets were washed three times with 1:1 ethanol:ethylacetate to remove free DNPH. After air drying, pellets were resuspended in 2× reducing Laemmli sample buffer. Proteins were separated by SDS-PAGE (10% Tris-HCl resolving gel) and transferred to polyvinylidene difluoride (PVDF) membrane. Derivatized proteins were detected by Western immunoblotting with anti-dinitrophenyl-KLH (anti-DNP) antibody. In particular, PVDF membrane was washed in PBST (10mM Na phosphate, pH 7.2, 0.9% (w/vol) NaCl, 0.1% (vol/vol) Tween-20) and blocked for 1 h in 5% (w/vol) nonfat dry milk in PBST. After washing three times with PBST for 5 min each, carbonyl formation was probed by 2 h incubation with 5% milk/PBST containing anti-DNP antibodies (1:40,000 dilution). After three 5-mins washes with PBST, the membrane was incubated in a 1:80,000 dilution of the secondary antibody linked to horseradish peroxidase in 5% milk/PBST for 1 h. After washing three times with PBST for 5 min each, immunostained protein bands were visualized with enhanced chemiluminescence detection. Densitometric analysis was performed after scanning the chemiluminescence films by using Image J 1.40d software (National Institutes of Health). A single assay for each sample was performed, so no intra- or inter-assay variation can be calculated.

Lipid peroxidation was measured according to the method developed by Ohkawa et al. (1979) and modified by Cinar et al. (2014) for blood samples. About 100 µL of blood were added to a solution composed by TCA 12%, thiobarbituric acid (TBA 0.37%) and Tris-HCl (0.6 M), and boiled for 1 hour. After a centrifugation at 11,500 rpm for 15 min at 4 °C, the absorbance of the obtained supernatant was measured at 535 nm and the amount of thiobarbituric acid reactive substances (TBARS) formed was calculated and expressed as nmol TBARS/µL. A single assay for each sample was performed, so no intra- or inter-assay variation can be calculated.

**Telomere length analysis**

Telomere length analysis was performed according to the method described by Parolini et al. (2015). Genomic DNA was extracted from 10-20 μl of red blood cells using 1 ml TNSE buffer (10 mM Tris HCl, 400 mM NaCl, 100 mM EDTA and 0.6% SDS) and a standard phenol/chloroform method. DNA samples of nestlings from the same nest were extracted in the same batch. We measured the quantity and purity of the extracted genomic DNA using a Nanophotometer (IMPLEN). Telomere length was measured by the monochrome multiplex quantitative PCR method (MMQPCR; Cawthon, 2009) on a PikoReal 96 thermal cycler (Thermo Scientific): telomere length was measured as the ratio (T/S) of the amount of telomeric repeats (T) to the amount of a single copy gene (S), relative to a reference sample. By this method, telomere length is evaluated indirectly by measuring the relative number of telomeric repeats in a genome and it is indicated from now as relative telomere length (RTL). The sequences of telomeric primers for MMQPCR were (telg 5’-ACACTAAGGTTTGGGTTTGGGTTTGGGTTTGGGTTAGTGT-3’; telc 5’-TGTTAGGTATCCCTATCCCTATCCCTATCCCTATCCCTAACA-3’), while the single copy sequence used as control was a fragment from the 12^th^ exon of the swallow CTCF gene (CCCTC-binding factor zinc finger protein). The CTCF primers used were: forward (5’-CCCGCGGCGGGCGGCGCGGGCTGGGCGGCTCCCAATGGAGACCTCAC-3’) and reverse (5’-CGCCGCGGCCCGCCGCGCCCGTCCCGCCCATCACCGGTCCATCATGC-3’); these primers are composed of a swallow genomic sequence and a GC-clamp at the 5’ end (underlined) to increase the melting temperature of the PCR product. Since the melting temperature of telomeric and CTCF PCR products are different, both primer pairs could be used in the same reaction. PCR reactions were prepared using 20 ng of genomic DNA as template, 1x DyNAmo ColorFlash SYBR Green qPCR Master Mix (Thermo Scientific), telomeric and CTCF primers at a final concentration of 1,000 nM and 500 nM each, respectively. Three-fold serial dilutions of a barn swallow reference sample (from 5.5 to 150 ng) were included in each plate to produce a standard curve to measure reaction efficiency and quantify the amount of telomeric repeats and single copy gene in each sample. We used the same reference sample per plate. All reactions were run in triplicate and six plates containing 25 samples each were performed. Cycling parameters for the PCR reactions were: Stage 1: 15 min at 95 °C; Stage 2: 2 cycles of 15s at 94 °C, 15s at 49 °C; and Stage 3: 35 cycles of 15s at 94 °C, 10s at 62 °C, 15s at 74 °C with signal acquisition, 10s at 84 °C, 15s at 88 °C with signal acquisition. The PikoReal Software (Thermo Scientific) was used to calculate the amount of telomeric repeats (T) for each sample by interpolation of the quantification Cycle (Cq) into the linear function y = ax + b of the standard curve of the telomeric primers. Similarly, the software calculates the amount of the single copy gene (S) for each sample. Mean values for T and S for each sample were used to calculate the T/S ratios relative to a reference sample, so telomere length was indicated as relative telomere length (RTL).

All reactions were run in triplicate and five plates containing on average 25 samples each were performed. Ten samples were run in each plate. The mean reaction efficiencies for both telomere and CTCF amplifications were greater than 84%. The intra- and inter-plate repeatability of RTL measures, expressed as intra-class correlation coefficient, was 0.56 and 0.59, respectively. The mean intra-and inter-plate coefficient of variation (± SD) of RTL measures was 10.9 ± 8.6 % and 15.5 ± 10.7 %, respectively.

Since the MMQPCR method evaluates the number of telomeric repeats, it cannot be used when large amounts of telomeric-like repeats at non-terminal sites (Interstial Telomeric Sequences, ITSs) are present in the genome under study. ITSs have been described in all vertebrate species analyzed so far and can be classified, according to sequence organization, into short-ITSs (s-ITSs), composed by short stretches of TTAGGG repeats (up to a few hundreds bp), and heterochromatic-ITSs (het-ITSs), composed by extended blocks of repeats spanning several kilobases and located mainly at pericentromeric regions (Ruiz-Herrera et al., 2008). Since the sequence of the yellow-legged gull genome was not available, a preliminary analysis aimed at determining the possible presence of het-ITSs was carried out by the standard Terminal Restriction Fragment (TRF) method (Figure S1). and by a *Bal*31 assay (Figure S2) as previously described (Smirnova et al., 2013; Faravelli et al., 2002; Parolini et al., 2015).

For TRF analysis, genomic DNA was digested with the restriction enzymes HinfI and RsaI (Thermo Scientific) separated by electrophoresis, denatured and transferred to a nylon membrane (Amersham Hybond-N, GE Healthcare). The DNA was then hybridized with a ^32^P-α[dCTP]-labeled telomeric probe and exposed to an autoradiographic film. As in barn swallows (Parolini et al 2015), no intense bands corresponding to het-ITSs were detected in yellow-legged gulls. As expected (Faravelli et al. 2002), in CHO cells several intense and discrete bands, corresponding to extended blocks of interstitial telomeric sequences, were observed (Figure S1).

For the Bal31 assay, genomic DNA was digested with either 0.05 (chicken DT40) or 0.005 (yellow-legged gull and barn swallow) units of *Bal*31 (Takara) per μg of DNA. Aliquots containing 3 μg of digested DNA were withdrawn from all reactions after 0, 5, 10 and 30 minutes. Additional aliquots of digested DT40 and barn swallow genomic DNAs were withdrawn after 60 (chicken DT40 and barn swallow) and 120 minutes (chicken DT40). Reactions were blocked by the addition of EGTA (final concentration 20 mM) and incubation at 65°C for 10 minutes. After phenol-chloroform extraction, DNAs were ethanol-precipitated, resuspended in water and digested for 12 hours with 10 units of *Hinf*I (Thermo Scientific) per μg of DNA. Digested DNA was electrophorased in 1% agarose gel, denatured and transferred to a nylon membrane (Amersham Hybond-N, GE Healthcare). Membranes were then hybridized with a ^32^P-α[dCTP]-labeled telomeric probe and exposed to autoradiography films. In DT40 we observed intense bands, resistant to *Bal*31 digestion, hybridizing with the telomeric repeat probe and corresponding to extended blocks of het-ITSs were located at internal chromosome sites, as expected. On the contrary, when we digested yellow-legged gull DNA with the *Bal*31 exonuclease, we detected a clear reduction in intensity and molecular weight of the smear, similar to that observed for barn swallow DNA, indicating that the majority of telomeric repeats detected in this sample were located at chromosome ends. This experiment suggests that, if present, ITSs in the yellow-legged gull genome are composed by a small number of repeats, as in human and barn swallow.


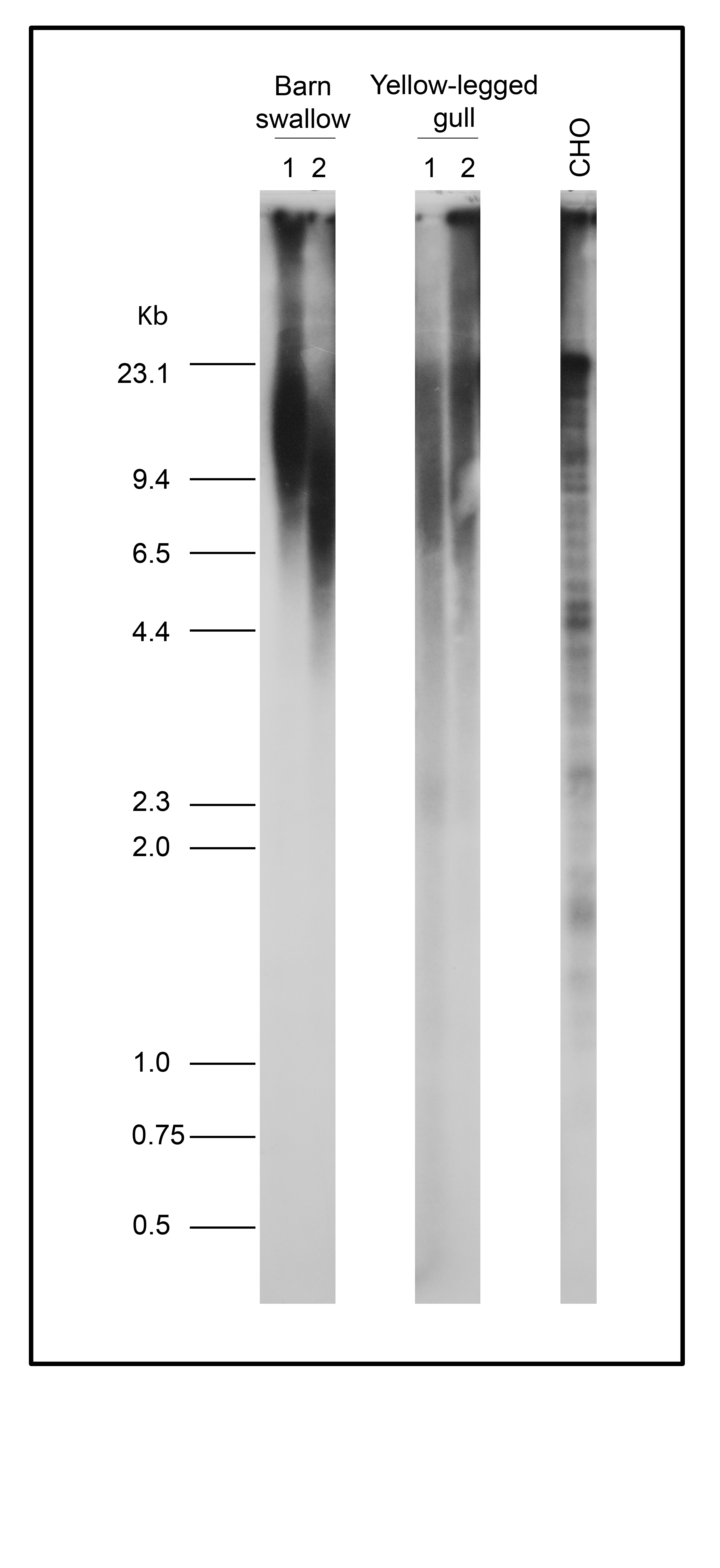


**Figure S1**. Terminal Restriction Fragment (TRF) analysis by Southern blotting in 2 barn swallows, 2 yellow-legged gulls and Chinese Hamster Ovary (CHO).

**
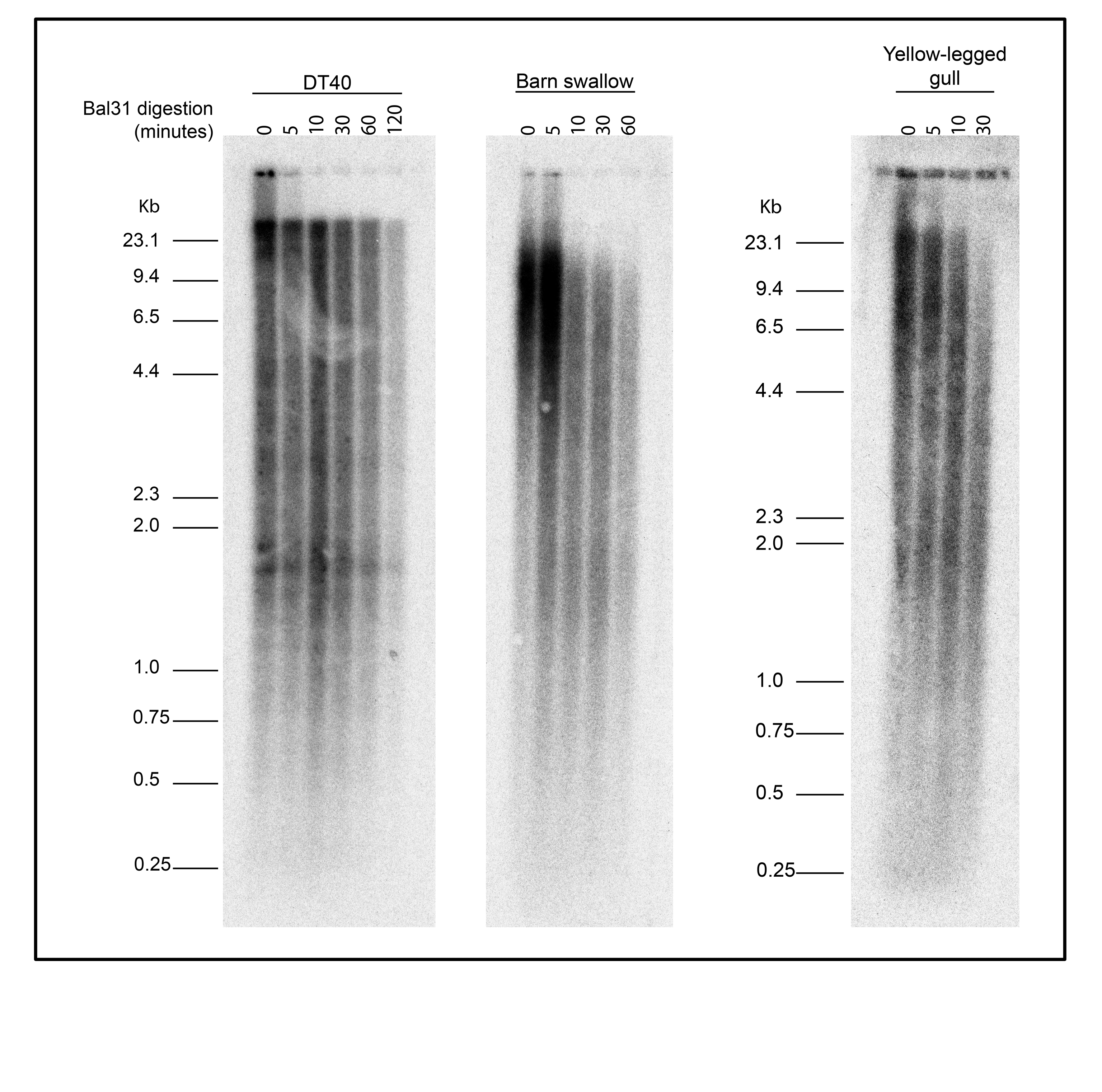
**

**Figure S2**. *Bal*31 exonuclease assay in chicken (DT40), barn swallow and yellow-legged gull.

**Analysis of Vitamin E content in yolk**

To assess the reliability of the injection procedure we first analyzed whether the concentration of VE in the residual yolk sac differed between sham- and VE-injected eggs. We relied on the yolk sac samples of 66 embryos (n = 26 nests) from a companion study in which we investigated potential effects of the injection of a physiological VE level on embryo traits. We highlight that the eggs were injected with the same VE concentrations described in the present study following the same experimental design. The concentration of VE in residual yolk sac was determined according to Karadas et al. (2006) using high-performance liquid chromatography system (Shimadzu Liquid Chromatography, LC-10AD, Japan Spectroscopic Co. Ltd.). Briefly, 100-150 mg of yolk were homogenized with 1 mL of ethanol plus 0.7 mL NaCl 5% and extracted twice by centrifugation with 2 mL of hexane each. Then, hexane extracts were pooled and evaporated at 60-65 °C under nitrogen flow and the residual was dissolved in 500 µL of dichloromethane:methanol mixture (50:50 v/v). VE (α- and γ-tocopherol) concentrations were detected with a Hypersil GOLD type 3µm C18 reverse-phase column (150 × 4.6 mm Phase Separation, Thermo Fisher Scientific 81, Wyman, Street Waltham, MA USA) with a mobile phase of methanol:distilled water (97:3 v/v) at a flow rate of 1.05 mL min^-1^ using fluorescence detection by excitation and emission wavelength of 295 nm and 330 nm, respectively. Peaks of α-, and γ-tocopherol were identified by comparison with the retention time of standards of tocopherols (Sigma, Poole, UK).

As expected, vitamin E concentration was significantly larger in vitamin E treated eggs compared to controls (F_1,44.5_ = 4.314; P = 0.044). Even if the effect of the laying sequence per treatment interaction on yolk sac vitamin E concentration was statically non-significant (F_2,47.8_ = 0.795; P = 0.457), the concentration of vitamin E in the residual yolk from VE-injected eggs was always higher compared to that measured in controls (Figure S3).

**Figure S3:** Mean of VE concentration (+SD) in residual yolk sac of yellow-legged gull eggs shortly before hatching (i.e. ‘cracking stage’, when eggshell fractures appear).

In detail, basing on data reported in Rubolini et al. (2011) in which levels of VE in eggs of yellow-legged gulls from the same colony have been measured, we expected a post-injection mean VE concentration in a-, b- and c-eggs as 145.6 µg/g (mean ± SD; 119.2 ± 26.4), 133 µg/g (mean ± SD; 104.9 ± 28.1) and 103.8 µg/g (mean ± SD; 81.4 ± 22.4), respectively, which corresponds on average to a 25% increase of VE concentration compared to the physiological levels of the species. Our analyses showed that VE concentration in residual yolk sac from VE-injected in a-, b- and c-eggs soon before hatching was 103.0 ± 26.8 µg/g, 97.5 ± 21.7 µg/g and 92.1 ± 18.6 µg/g (mean ± SD), respectively, showing on average a 25%, 6% and 18% increase (mean 16%) with respect to the corresponding sham-injected eggs. In addition, as we weighted the residual yolk sac in eggs at the cracking stage, we estimated the total yolk weight at the time of laying for each egg and, consequently the percentage of the residual yolk that each embryo has to adsorb before hatching. Then, we estimated the possible amount of VE at the deposition for each egg according to the percentage of the residual yolk at the cracking stage. Mean estimated total VE concentration in VE-injected a-, b- and c-eggs should 130 µg/g, 121 µg/g and 113 µg/g, which on average correspond to a 22% increase compared to the corresponding sham-injected eggs. Thus, the estimated VE concentrations based on our analysis are very close to the expected values by the study of Rubolini et al. (2011) and our data definitively confirm the effectiveness of VE-injection in the yolk of yellow-legged gull eggs.

Unfortunately, we cannot measure the concentration of VE in blood of hatchlings because of sample scarceness. However, since VE is efficiently transferred from yolk to developing embryos, we relied on the embryos developed into the eggs used to certify the injection methods (see above) and we certified VE transfer to the embryos by measuring the concentration of this antioxidant in two organs, namely the brain and the liver. Even if the concentrations of VE measured in VE–treated embryos soon before hatching were not significantly larger than controls, neither in the brain (F_1,59_ = 1.707; P = 0.196) nor in the liver (F_1,35_ = 0.305; P = 0.584), overall the measured concentrations were higher in chick organs from VE-injected eggs compared to sham-injected ones (Figure S4), revealing a higher transfer from yolk to organs in embryos from VE-injected eggs compared to controls.


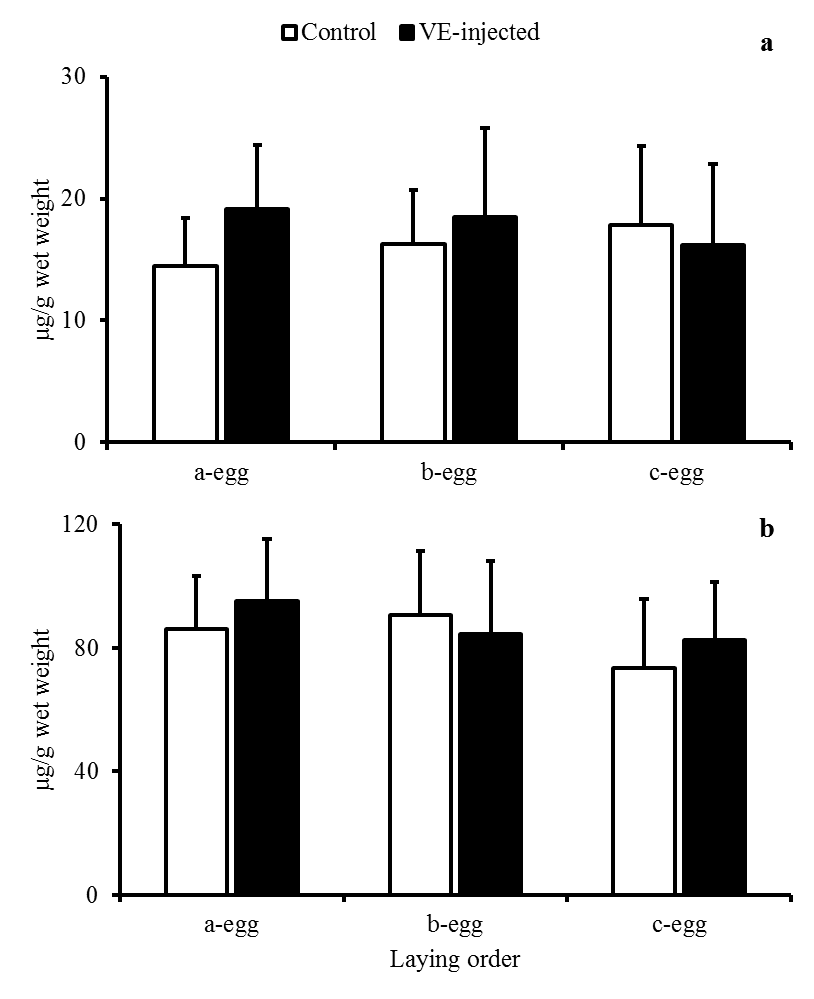


**Figure S4:** Mean of VE concentration (+SD) in brain (a) and liver (b) of yellow-legged gull embryos shortly before hatching (i.e. ‘cracking stage’, when eggshell fractures appear).

**References**

Cawthon RM. 2009 Telomere length measurement by a novel monochrome multiplex quantitative PCR method. *Nucleic Acids Res* **37**:e21. (doi: 10.1093/nar/gkn1027)

Cinar M, Yildirim E, Yigit AA, Yalcinkaya I, et al. 2014 Effects of dietary supplementation with vitamin C and vitamin E and their combination on growth performance, some biochemical parameters, and oxidative stress induced by copper toxicity in broilers. *Biol. Trace Elem. Res.* **158**, 186-196. (doi: 10.1007/s12011-014-9926-6)

Erel O. 2004 A novel automated direct measurement method for total antioxidant capacity using a new generation, more stable ABTS radical cation. *Clin. Biochem*. **37**, 277-285. (doi: 10.1016/j.clinbiochem.2003.11.015)

Erel O. 2005 A new automated colorimetric method for measuring total oxidant status. *Clin. Biochem*. **38**, 1103-1111. (doi: 10.1016/j.clinbiochem.2005.08.008)

Faravelli M, Azzalin CM, Bertoni L, Chernova O, Attolini C, Mondello C, Giulotto E. 2002 Molecular organization of internal telomeric sequences in Chinese hamster chromosomes. *Gene*. **283**: 11-16. (10.1016/S0378-1119(01)00877-0)

Karadas F, Grammenidis E, Surai PF, Acamovic T, Sparks NHC. 2006 Effects of carotenoids from lucerne, marigold and tomato on egg yolk pigmentation and carotenoid composition. *Br. Poult. Sci.* **47**, 561-566. (doi: 10.1080/00071660600962976)

Ohkawa H, Ohishi N, Yagi K. 1979 Assay for lipid peroxides in animal tissues by thiobarbituric acid reaction. *Anal. Biochem*. **95**, 351-358. (doi: 10.1016/0003-2697(79)90738-3)

Parolini M, Colombo G, Valsecchi S, Mazzoni M, Possenti CD, et al. 2016 Potential toxicity of environmentally relevant perfluorooctane sulfonate (PFOS) concentrations to yellow-legged gull *Larus michahellis* embryos. *Environ. Sci. Pollut. Res.* **23**, 426-437. (doi: 10.1007/s11356-015-5248-2)

Parolini M, Romano A, Khoriauli L, Nergadze SG, Caprioli M, et al. 2015 Early-life telomere dynamics differ between the sexes and predict growth in the barn swallow (*Hirundo rustica*). *PLoS ONE* **10**:e0142530. (doi: 10.1371/journal.pone.0142530)

Parolini M, Romano M, Caprioli M, Rubolini D, Saino N. 2015 Vitamin E deficiency in last-laid eggs limits growth of yellow-legged gull chicks. *Funct. Ecol.* **29**, 1070-1077. (doi: 10.1111/1365-2435.12412)

Romano M, Caprioli M, Ambrosini R, Rubolini D, Fasola M, Saino N. 2008 Maternal allocation strategies and differential effects of yolk carotenoids on the phenotype and viability of yellow-legged gull (*Larus michahellis*) chicks in relation to sex and laying order. *J. Evol. Biol.* **21**, 1626–1640. (doi: 10.1111/j.1420-9101.2008.01599.x)

Rubolini D, Romano M, Martinelli R, Saino N. 2006 Effects of elevated yolk testosterone levels on survival, growth and immunity of male and female yellow-legged gull chicks. *Behav. Ecol. Sociobiol.* **59**, 344-352. (doi: 10.1007/s00265-005-0057-0)

Rubolini D, Romano M, Navara KJ, Karadas F, Ambrosini R, et al. 2011 Maternal effects mediated by egg quality in the Yellow-legged Gull *Larus michahellis* in relation to laying order and embryo sex. *Front. Zool.* **8**, 24. (doi: 10.1186/1742-9994-8-24)

Ruiz-Herrera A, Nergadze SG, Santagostino M, Giulotto E. 2008Telomeric repeats far from the ends: mechanisms of origin and role in evolution. *Cytogenet. Genome Res*. **122**, 219-228. (doi: 10.1159/000167807)

Smirnova A, Gamba R, Khoriauli L, Vitelli V, Nergadze SG, Giulotto E. 2013 TERRA expression levels do not correlate with telomere length and radiation sensitivity in human cancer cell lines. *Front. Oncol*. **10**, 115. (doi: 10.3389/fonc.2013.00115)
